# Supplementary material for: Association between carbohydrate quality and glycemic control in individuals with type 1 diabetes: A cross-sectional and meal-based analysis
Source: PLoS One. 2026 Feb 2;21(2):e0341915. doi: 10.1371/journal.pone.0341915 (PMC12863503; doi:10.1371/journal.pone.0341915)
Supplement: S1 File — Supporting information. Contains: S1 Fig. Distribution of the daily macro-nutrient proportions derived from the total energy intake; S2 Fig. Box-plot illustrating the correlation between carbohydrate (%E) intake and postprandial blood glucose AUC; S1 Table. Baseline characteristics of individuals with T1D in FFQ cohort; S2 Table. Characteristics of nutrient intake and indicators for glycemic control derived from CGM in 3-day food records; S3 Table. Missing values per variable (FFQ cohort, n = 155); S4 Table. Missing values per variable (3d24h dietary record cohort, number of days = 188); S5 Table. Missing values per variable (3d24h dietary record cohort, number of lunch = 188). (DOCX) [file pone.0341915.s001.docx]

**Supporting information**

**S1 File**


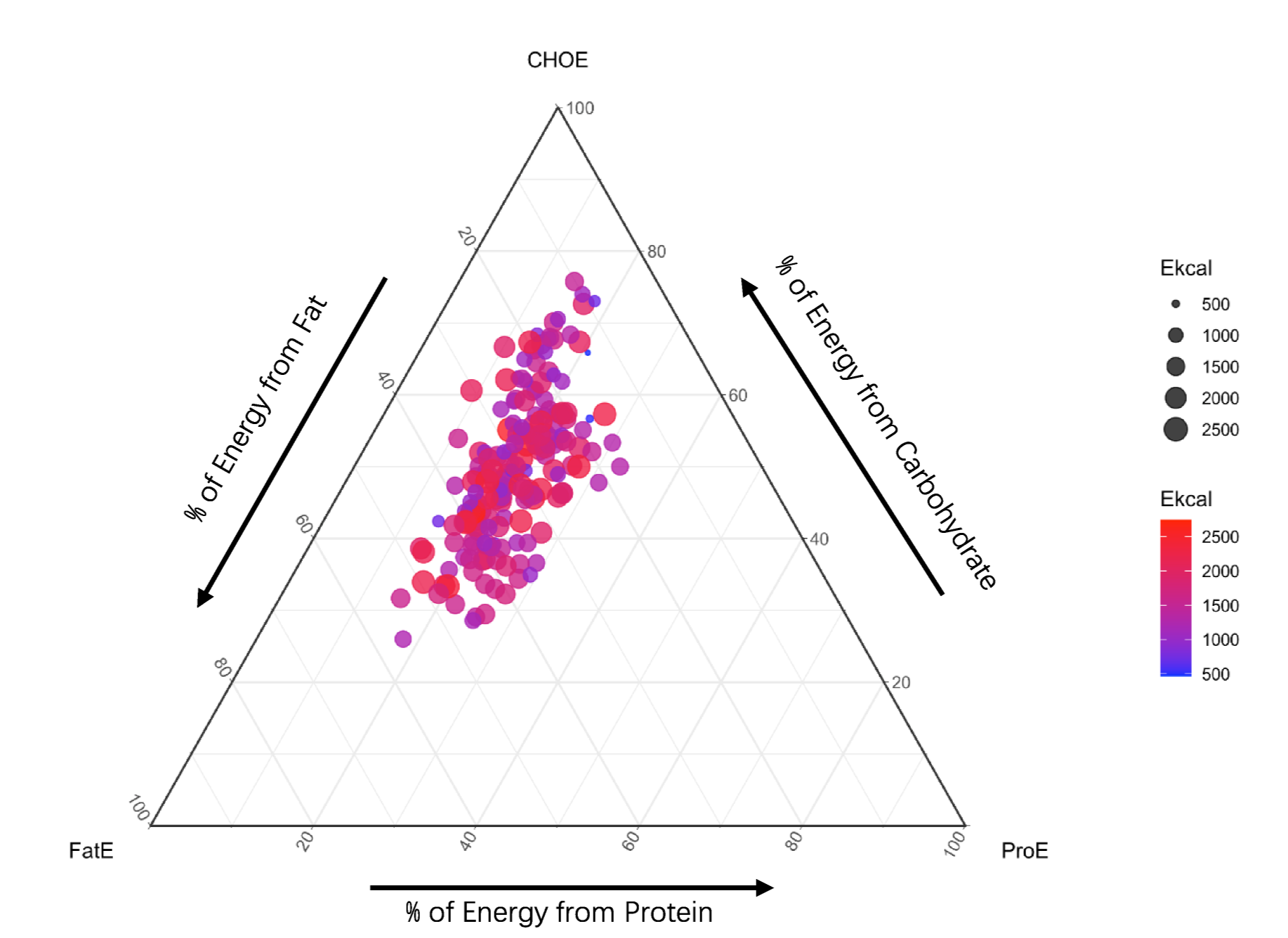


**S1 Fig. Distribution of the daily macro-nutrient proportions derived from the total energy intake for individuals in 3-day food dietary cohort**

Each daily macro-nutrient intake represented by a single point


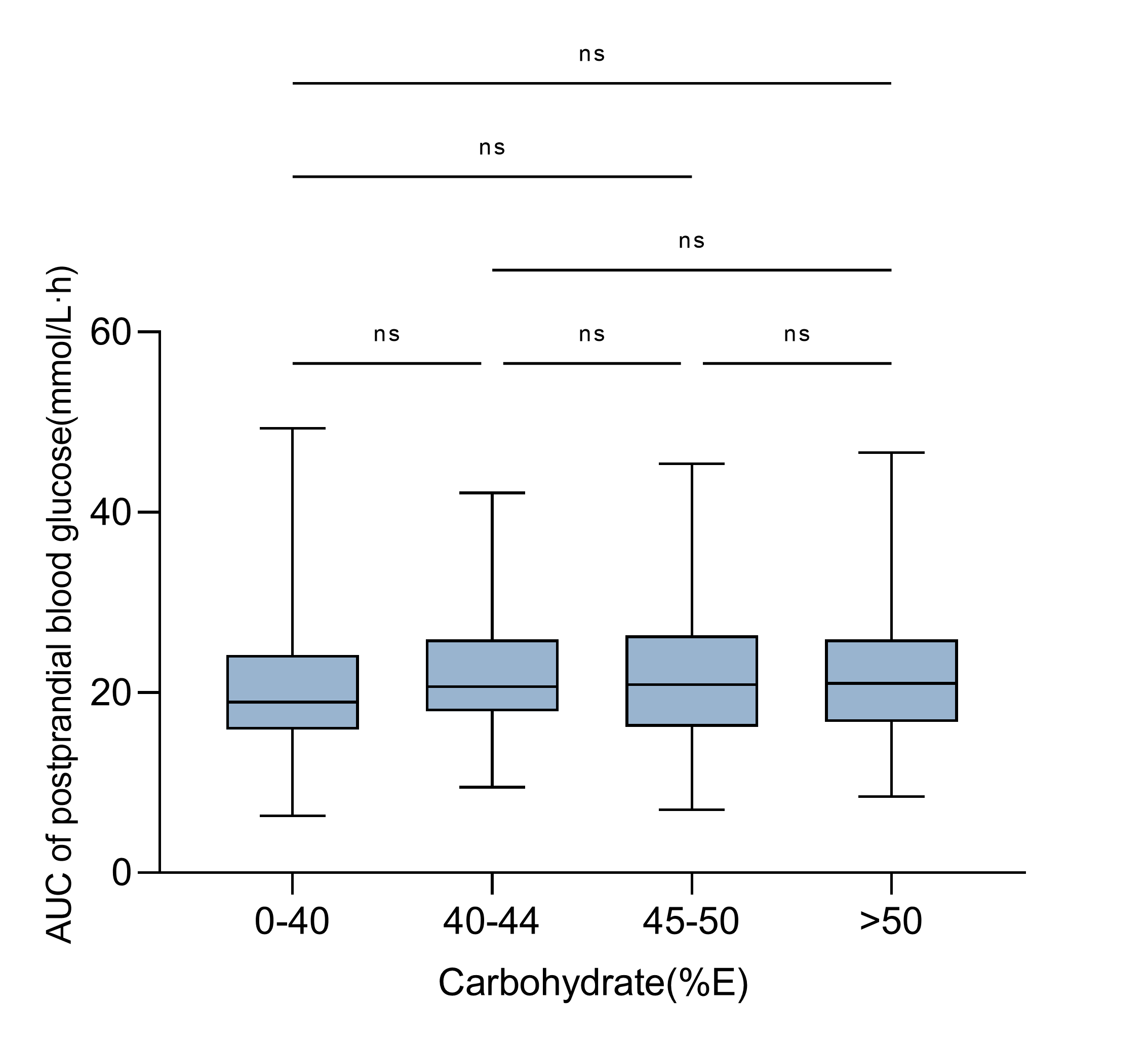


**S2 Fig. Box-plot illustrating the correlation between carbohydrate (%E) intake and postprandial blood glucose AUC.**

Boxes show the 25th percentile (bottom of box), median (box midline), and 75th percentile (top of box). The error bars show the distribution.

**S1 Table. Baseline characteristics of individuals with T1D in FFQ cohort**

| **Characteristic** | **T1D patients (N=155)** |
| --- | --- |
| Age (years) | 20.00 [14.00, 27.00] |
| Gender, male/female | 65/90 |
| Duration (years) | 5.00 [3.02, 9.94] |
| BMI (kg/m^2) | 20.7 (3.45) |
| Insulin dose (IU) | 35.00 [23.50, 46.50] |
| Insulin dose (IU/kg) | 0.63 [0.44, 0.78] |
| Insulin pump/ non-insulin pump | 62/93 |
| Physical activity (MET-min/wk) | 4350.00 [3555.00, 5107.50] |
| HbA1c (%) | 6.68 [6.16, 7.26]] |

Data are presented as mean (SD) for continuous variables with normal distribution, median [interquartile range] for continuous variables that were skewed.

**S2 Table. Characteristics of nutrient intake and indicators for glycemic control derived from CGM in 3-day food records**

| **Variables** | **Total** |
| --- | --- |
| **Demographic characteristics** | |
| Number of days | 188 |
| Age (years) | 20.00 [16.00, 30.00] |
| Duration (years) | 5.02 [3.14, 7.58] |
| BMI (kg/m^2) | 20.83 [18.36, 22.60] |
| Insulin dose (IU/kg) | 0.69 [0.41, 0.83] |
| Insulin pump/Non-insulin pump | 56 /132 |
| Physical activity (MET-min/wk) | 4750.00 [3960.00, 5888.00] |
| **Meal composition** | |
| Carbs, %E | 48.24 (10.84) |
| Fat, %E | 29.00 [21.00, 37.00] |
| Protein, %E | 19.00 [16.00, 22.60] |
| Fiber, g/d | 8.62 [5.59, 12.19] |
| Glycemic index, % | 58.53 [52.09, 65.29] |
| Glycimic load, units | 105.99 [79.74, 138.91] |
| Whole-grain, %CHO | 16.00 [0.00, 42.75] |
| Refined-grain, %CHO | 57.50 [30.75, 75.25] |
| Other sources, %CHO | 21.00 [13.25, 35.75] |
| **Glycemic measures in CGM** | |
| TIR | 88.70 [73.35, 97.35] |
| SD | 1.70 [1.10, 2.38] |
| LAGE | 7.40 [5.20, 9.47] |
| MAGE | 4.32 [2.80, 6.22] |

Data are presented as mean (SD) for continuous variables with normal distribution, median [interquartile range] for continuous variables that were skewed, and frequency (%) for categorical variables

**S3 Table. Missing values per variable (FFQ cohort, n=155)**

| variable | Missing values (n) | Missing values (%) |
| --- | --- | --- |
| Age (years) | 0 | 0 |
| Duration (years) | 0 | 0 |
| BMI (kg/m^2) | 0 | 0 |
| Insulin dose (IU | 0 | 0 |
| Insulin dose (IU/kg) | 0 | 0 |
| Insulin regimen | 0 | 0 |
| Physical activity (MET-min/wk) | 0 | 0 |
| HbA1c | 0 | 0 |
| Carbs, %E | 0 | 0 |
| Fat, %E | 0 | 0 |
| Protein, %E | 0 | 0 |
| Fiber, g/d | 0 | 0 |
| Refined-grain, g/d | 0 | 0 |
| Whole-grain, g/d | 0 | 0 |

**S4 Table. Missing values per variable (3d24h dietary record cohort, number of days=188)**

| Variable | Missing values (n) | Missing values (%) |
| --- | --- | --- |
| Age (years) | 0 | 0 |
| Duration (years) | 0 | 0 |
| BMI (kg/m^2) | 0 | 0 |
| Insulin dose (IU) | 0 | 0 |
| Insulin dose (IU/kg) | 0 | 0 |
| Insulin regimen | 0 | 0 |
| Physical activity (MET-min/wk) | 0 | 0 |
| Carbs, %E | 0 | 0 |
| Fat, %E | 0 | 0 |
| Protein, %E | 0 | 0 |
| Fiber, g/d | 0 | 0 |
| Glycemic index, % | 0 | 0 |
| Glycimic load, units | 0 | 0 |
| Whole-grain, %CHO | 2 | 1.1 |
| Refined-grain, %CHO | 2 | 1.1 |
| TIR | 2 | 1.1 |
| SD | 2 | 1.1 |
| LAGE | 2 | 1.1 |
| MAGE | 2 | 1.1 |

**S5 Table. Missing values per variable (3d24h dietary record cohort, number of lunch=188)**

| Variable | Missing values (n) | Missing values (%) |
| --- | --- | --- |
| Age (years) | 0 | 0 |
| Duration (years) | 0 | 0 |
| BMI (kg/m^2) | 0 | 0 |
| Insulin dose (IU) | 0 | 0 |
| Insulin dose (IU/kg) | 0 | 0 |
| Insulin regimen | 0 | 0 |
| Physical activity (MET-min/wk) | 0 | 0 |
| Carbs, %E | 0 | 0 |
| Fat, %E | 0 | 0 |
| Protein, %E | 0 | 0 |
| Fiber, g/d | 0 | 0 |
| Glycemic index, % | 0 | 0 |
| Glycimic load, units | 0 | 0 |
| Whole-grain, %CHO | 0 | 0 |
| Refined-grain, %CHO | 0 | 0 |
| TIR | 0 | 0 |
| SD | 0 | 0 |
| LAGE | 0 | 0 |
| MAGE | 44 | 23.4 |
