# Supplementary material for: Association between carbohydrate quality and glycemic control in individuals with type 1 diabetes: A cross-sectional and meal-based analysis
Source: PLoS One. 2026 Feb 2;21(2):e0341915. doi: 10.1371/journal.pone.0341915 (PMC12863503; doi:10.1371/journal.pone.0341915)
Supplement: Attachment — Submitted filename: Response to reviewers.docx [file pone.0341915.s004.docx]

Response to comments

We sincerely thank the editor and all reviewers for their valuable feedback that we have used to improve the quality of our manuscript. The reviewers’ comments are laid out below in italicized font and specific concerns have been numbered. Our response is given in red font and changes to the manuscript are highlighted in yellow. The highlighted modified sections in the text correspond to the line numbers of the ***Revised Manuscript (clean copy)*** version.

***Reviewer #1:***

***Required Revisions:***

1. *All conclusions must be reframed using associational rather than causal language.*

Response 1: Following the reviewer's suggestions, we have implemented several revisions in both the abstract and main text, as detailed below:

In the abstract section, we have replaced the original statement “Increasing the consumption of dietary fiber is beneficial for achieving long-term glycemic control. Additionally, increasing the intake of whole grains, especially at lunch, as a source of high-quality carbohydrates, is helpful in reducing glycemic fluctuations, which is of crucial significance for reducing the risk of long-term complications in T1D patients.” with “From a long-term glycemic control perspective, higher dietary fiber intake appears to be associated with improved HbA1c levels, while in terms of short-term glycemic variability, increased whole grain consumption is associated with reduced glucose fluctuations. However, as a cross-sectional analysis, these findings represent observational associations rather than causal evidence. Further validation through prospective cohorts and randomized trials is needed to assess clinical applicability.” (Line 38-43) to present a more balanced conclusion.

In the result session, we have replaced the original statement “Increasing the proportion of whole grains in the source of carbohydrates is beneficial for reducing variations in postprandial blood glucose levels” with “A higher proportion of whole grains in carbohydrate sources is associated with reduced postprandial glucose variability”. (Line 251-252)

In the discussion section, we have replaced the original statement “and incorporate a higher proportion of whole grains in carbohydrate sources may be beneficial for reducing postprandial glycemic fluctuations, offering potential guidance for dietary choices in this population” with “and incorporate a higher proportion of whole grains in carbohydrate sources may be associated with reduced postprandial glycemic fluctuations” (Line 325-326), and we have replaced the original statement “The clinical significance of this study lies in the discovery that dietary fiber intake is beneficial for long-term glycemic control and contributes to blood glucose compliance (HbA1c≤6.5%) in T1D patients” with “This study clinically demonstrates significant associations between dietary fiber intake and both long-term glycemic control and HbA1c target attainment (≤6.5%) in T1D patients.” (Line 376-377) , and have replaced the original statement “For patients with T1D, increasing the consumption of whole grains, particularly at lunch, as a source of high-quality carbohydrates, is advantageous for reducing post-meal glycemic fluctuations when a low-carbohydrate diet pattern is not required” with “For patients with T1D, higher consumption of whole grains as a primary carbohydrate source - particularly during lunch - is associated with attenuated postprandial glycemic excursions when a low-carbohydrate diet pattern is not required.” (Line 382-384)

In the conclusion section, we have replaced the original statement “Increasing the consumption of dietary fiber is beneficial for achieving long-term glycemic control. Additionally, increasing the intake of whole grains, especially at lunch, as a source of high-quality carbohydrates, is helpful in reducing post-meal glycemic fluctuations, which is of crucial significance for reducing the risk of long-term complications in T1D patients” with “Evidence from this study indicates that elevated dietary fiber intake is significantly associated with enhanced long-term glycemic control in individuals with T1D. Notably, our findings reveal that preferential consumption of whole grains as the predominant carbohydrate source, with particular emphasis on lunchtime intake, is correlated with reduced postprandial glucose variability.” (Line 408-411)

2. *Effect sizes with confidence intervals must be reported for all key findings.*

Response 2: Following the reviewer's suggestions, as shown in Table 2, Table 4 and Table 6, we have reported the standardized regression coefficients with 95% confidence intervals to facilitate effect size comparison. In the revised table, we replaced the unstandardized raw regression coefficients (Est.) with β values.

1) the original Table 2:

Table 2. Associations between meal nutrient composition and HbA1c levels

| **Meal composition** | **Crude model** | | | **Model 1** | | | **Model 2** | | |
| --- | --- | --- | --- | --- | --- | --- | --- | --- | --- |
|  | Est. | SE | p value | Est. | SE | p value | Est. | SE | p value |
| Carbs, E% | -2.134 | 1.103 | 0.055 | 2.039 | 1.389 | 0.144 | 2.390 | 1.451 | 0.102 |
| Protein, E% | 6.213 | 3.271 | 0.059 | 12.709 | 3.941 | 0.002** | 11.415 | 3.995 | 0.005** |
| Fat, E% | 0.342 | 1.284 | 0.790 | -2.910 | 1.304 | 0.027* | -3.328 | 1.354 | 0.015* |
| Fiber, g/d | -0.057 | 0.015 | <0.001** | -0.058 | 0.020 | 0.004** | -0.060 | 0.019 | 0.002** |

Multivariate linear regression; *p<0.05; **p<0.01

Model 1 adjusted for other macro-nutrients listed.

Model 2, as for Model 1, and adjusted for age, duration, BMI, total insulin dose, insulin regimen and physical activity.

the revised Table 2:

Table 2. Associations between meal nutrient composition and HbA1c levels

| **Meal composition** | **Crude model** | | **Model 1** | | **Model 2** | |
| --- | --- | --- | --- | --- | --- | --- |
|  | β  (95% CI) | *p* value | β  (95% CI) | *p* value | β  (95% CI) | *p* value |
| Carbs, E% | -0.16  (-0.18, -0.13) | 0.06 | 0.15  (0.12, 0.18) | 0.14 | 0.17  (0.14, 0.20) | 0.10 |
| Protein, E% | 0.15  (0.09, 0.22) | 0.06 | 0.27  (0.21, 0.34) | <0.01** | 0.23  (0.17, 0.30) | <0.01** |
| Fat, E% | 0.02  (-0.04, 0.05) | 0.79 | -0.18  (-0.21, -0.16) | 0.03* | -0.21  (-0.24, -0.18) | 0.02* |
| Fiber, g/d | -0.30  (-0.33, -0.27) | <0.01** | -0.31  (-0.34, -0.27) | <0.01** | -0.32  (-0.35, -0.28) | <0.01** |

Multivariate linear regression; *p<0.05; **p<0.01

Model 1 adjusted for other macro-nutrients listed.

Model 2, as for Model 1, and adjusted for age, duration, BMI, total insulin dose, insulin regimen and physical activity.

2) the original Table 4:

Table 4. Associations between meal nutrient composition and glycemic measures in CGM

| **Meal composition** | **model** | **TIR** | | **SD** | | **LAGE** | | **MAGE** | |
| --- | --- | --- | --- | --- | --- | --- | --- | --- | --- |
|  |  | Est. | *p* value | Est. | *p* value | Est. | *p* value | Est. | *p* value |
| Fat, %E | 1 | 22.12 | 0.06 | -1.21 | 0.03* | -3.51 | 0.07 | -1.69 | 0.31 |
|  | 2 | 23.01 | 0.04 | -1.23 | 0.02* | -3.04 | 0.12 | -1.54 | 0.36 |
| Protein, %E | 1 | 16.20 | 0.37 | -1.36 | 0.12 | -4.89 | 0.11 | -3.64 | 0.19 |
|  | 2 | 16.88 | 0.33 | -1.10 | 0.19 | -3.82 | 0.20 | -3.40 | 0.21 |
| Carbs, %E | 1 | 15.08 | 0.31 | 0.11 | 0.88 | -0.78 | 0.75 | -2.35 | 0.29 |
|  | 2 | 16.02 | 0.27 | 0.04 | 0.96 | -1.17 | 0.63 | -2.45 | 0.25 |
| Fiber, g/d | 1 | 0.37 | 0.07 | -0.04 | <0.01** | -0.13 | <0.01** | -0.08 | <0.01** |
|  | 2 | 0.37 | 0.06 | -0.04 | <0.01** | -0.13 | <0.01** | -0.09 | <0.01** |
| Glycemic Index, % | 1 | -0.07 | 0.47 | 0.01 | 0.03* | 0.03 | 0.11 | 0.03 | 0.06 |
|  | 2 | -0.06 | 0.55 | 0.01 | 0.03* | 0.03 | 0.11 | 0.02 | 0.13 |
| Glycemic Load, units | 1 | -0.03 | 0.39 | 0.01 | 0.01* | 0.01 | 0.13 | 0.00 | 0.42 |
|  | 2 | -0.03 | 0.50 | 0.00 | 0.02* | 0.01 | 0.27 | 0.00 | 0.99 |
| Whole-grain, %CHO | 1 | 12.96 | 0.01* | -0.79 | <0.01** | -2.29 | 0.01* | -1.65 | 0.02* |
|  | 2 | 12.19 | 0.01* | -0.83 | <0.01** | -2.40 | <0.01** | -1.47 | 0.04* |
| Refined-grain, %CHO | 1 | -11.96 | 0.01* | 0.71 | <0.01** | 2.26 | <0.01** | 1.48 | 0.02* |
|  | 2 | -11.27 | 0.01* | 0.69 | <0.01** | 2.16 | <0.01** | 1.41 | 0.02* |

* *p*<0.05；***p*<0.01；mix-effect model；

Model 1 adjusted for other macro-nutrients listed.;

Model 2, as for Model 1, and adjusted for age, duration, BMI, total insulin dose, Insulin regimen and physical activity. TIR, Time in range; SD, Standard Deviation; LAGE, Largest Amplitude of Glycemic Excursions; MAGE, Mean Amplitude of Glycemic Excursions. Whole-grain, %CHO, the proportion of refined grains in the carbohydrate source; Refined-grain, %CHO, the proportion of refined grains in the carbohydrate source.

the revised Table 4:

Table 4. Associations between meal nutrient composition and glycemic measures in CGM

| **Meal composition** | **model** | **TIR** | | **SD** | | **LAGE** | | **MAGE** | |
| --- | --- | --- | --- | --- | --- | --- | --- | --- | --- |
|  |  | β  (95% CI) | *p* value | β  (95% CI) | *p* value | β  (95% CI) | *p* value | β  (95% CI) | *p* value |
| Fat, %E | 1 | 0.12  (-0.01, 0.24) | 0.06 | -0.15  (-0.29, -0.02) | 0.03* | -0.12  (-0.25, 0.01) | 0.07 | -0.08  (-0.22, 0.07) | 0.31 |
|  | 2 | 0.12  (0.001, 0.24) | 0.04 | -0.15  (-0.29, -0.02) | 0.02* | -0.10  (-0.24, 0.03) | 0.12 | -0.07  (-0.22, 0.08) | 0.36 |
| Protein, %E | 1 | 0.05  (-0.06, 0.15) | 0.37 | -0.08  (-0.21, 0.02) | 0.12 | -0.09  (-0.20, 0.02) | 0.11 | -0.09  (-0.22, 0.04) | 0.19 |
|  | 2 | 0.05  (-0.05, 0.15) | 0.33 | -0.09  (-0.19, 0.04) | 0.19 | -0.07  (-0.19, 0.04) | 0.20 | -0.08  (-0.22, 0.05) | 0.21 |
| Carbs, %E | 1 | 0.08  (-0.08, 0.24) | 0.31 | 0.01  (-0.16, 0.19) | 0.88 | -0.03  (-0.20, 0.14) | 0.75 | -0.11  (-0.31, 0.09) | 0.29 |
|  | 2 | 0.09  (-0.07, 0.24) | 0.27 | 0.005  (-0.17, 0.18) | 0.96 | -0.04  (-0.21, 0.13) | 0.63 | -0.11  (-0.31, 0.08) | 0.25 |
| Fiber, g/d | 1 | 0.12  (-0.01, 0.24) | 0.07 | -0.263  (-0.40, -0.13) | <0.01** | -0.25  (-0.38, -0.12) | <0.01** | -0.22  (-0.37, -0.07) | <0.01** |
|  | 2 | 0.12  (-0.01, 0.24) | 0.06 | -0.26  (-0.40, -0.13) | <0.01** | -0.25  (-0.39, -0.12) | <0.01** | -0.23  (-0.38, -0.08) | <0.01** |
| Glycemic Index, % | 1 | -0.04  (-0.15, 0.07) | 0.47 | 0.14  (0.02, 0.26) | 0.03* | 0.10  (-0.02, 0.21) | 0.11 | 0.13  (-0.01, 0.27) | 0.06 |
|  | 2 | -0.03  (-0.14, 0.07) | 0.55 | 0.14  (0.02, 0.26) | 0.03* | 0.10  (-0.02, 0.21) | 0.11 | 0.11  (-0.03, 0.25) | 0.13 |
| Glycemic Load, units | 1 | -0.07  (-0.25, 0.10) | 0.39 | 0.26  (0.07, 0.45) | 0.01* | 0.14  (-0.04, 0.34) | 0.13 | 0.09  (-0.12, 0.30) | 0.42 |
|  | 2 | -0.06  (-0.23, 0.11) | 0.50 | 0.23  (0.04, 0.43) | 0.02* | 0.10  (-0.08, 0.29) | 0.27 | 0.01  (-0.21, 0.23) | 0.99 |
| Whole-grain, %CHO | 1 | 0.16  (0.04, 0.28) | 0.01* | -0.23  (-0.36, -0.10) | <0.01** | -0.18  (-0.31, -0.05) | 0.01* | -0.17  (-0.32, -0.03) | 0.02* |
|  | 2 | 0.15  (0.03, 0.27) | 0.01* | -0.24  (-0.38, -0.11) | <0.01** | -0.19  (-0.32, -0.06) | <0.01** | -0.15  (-0.30, -0.01) | 0.04* |
| Refined-grain, %CHO | 1 | -0.17  (-0.29, -0.05) | 0.01* | 0.24  (0.11, 0.37) | <0.01** | 0.21  (0.08, 0.34) | <0.01** | 0.18  (0.03, 0.33) | 0.02* |
|  | 2 | -0.16  (-0.28, -0.05) | 0.01* | 0.23  (0.10, 0.37) | <0.01** | 0.20  (0.07, 0.33) | <0.01** | 0.17  (0.02, 0.32) | 0.02* |

* *p*<0.05；***p*<0.01；mix-effect model；

Model 1 adjusted for other macro-nutrients listed.;

Model 2, as for Model 1, and adjusted for age, duration, BMI, total insulin dose, Insulin regimen and physical activity. TIR, Time in range; SD, Standard Deviation; LAGE, Largest Amplitude of Glycemic Excursions; MAGE, Mean Amplitude of Glycemic Excursions. Whole-grain, %CHO, the proportion of refined grains in the carbohydrate source; Refined-grain, %CHO, the proportion of refined grains in the carbohydrate source.

3) the original Table 6:

Table 6. The associations between dietary factors and postprandial glycemic variability at lunch

| Meal composition | TIR | | SD | | LAGE | | MAGE | |
| --- | --- | --- | --- | --- | --- | --- | --- | --- |
|  | Est. | *p* value | Est. | *p* value | Est. | *p* value | Est. | *p* value |
| Fat, %E | 0.22 | 0.06 | -0.01 | 0.07 | -0.02 | 0.08 | -0.02 | 0.10 |
| Protein, %E | 0.12 | 0.41 | -0.01 | 0.34 | -0.01 | 0.40 | -0.02 | 0.21 |
| Carbs, %E | -0.12 | 0.24 | 0.00 | 0.41 | 0.01 | 0.55 | 0.00 | 0.74 |
| Fiber, g/d | 0.33 | 0.50 | -0.06 | <0.01** | -0.19 | <0.01** | -0.12 | 0.01* |
| Glycemic Index, % | -0.15 | 0.21 | 0.01 | <0.01** | 0.04 | <0.01** | 0.03 | 0.01* |
| Glycemic Load, units | -0.16 | 0.10 | 0.01 | 0.01* | 0.03 | 0.01* | 0.02 | <0.01** |
| Whole-grain,  %Carbs | 4.44 | 0.39 | -0.45 | 0.02* | -1.52 | 0.01* | -1.39 | <0.01** |
| Refined-grain,  %Carbs | -4.28 | 0.35 | 0.28 | 0.09 | 1.02 | 0.05 | 1.37 | <0.01** |

* *p*<0.05；***p*<0.01；

Mix-effect model.

Considering the participant’s ID as random effect and day as a nested random effect.

Adjusted for age, duration, BMI, total insulin dose, Insulin regimen, physical activity and other macro-nutrients listed.

The revised Table 6:

Table 6. The associations between dietary factors and postprandial glycemic variability at lunch

| **Meal composition** | **TIR** | | **SD** | | **LAGE** | | **MAGE** | |
| --- | --- | --- | --- | --- | --- | --- | --- | --- |
|  | β  (95% CI) | *p* value | β  (95% CI) | *p* value | β  (95% CI) | *p* value | β  (95% CI) | *p* value |
| Fat, %E | 0.14  (-0.01, 0.28) | 0.06 | -0.13  (-0.27, 0.01) | 0.07 | -0.12  (-0.26, 0.01) | 0.08 | -0.14  (-0.31, 0.02) | 0.10 |
| Protein, %E | 0.05  (-0.07, 0.18) | 0.41 | -0.06  (-0.20, 0.07) | 0.34 | -0.06  (-0.19, 0.07) | 0.40 | -0.10  (-0.26, 0.05) | 0.21 |
| Carbs, %E | -0.08  (-0.22, 0.05) | 0.24 | 0.06  (-0.08, 0.20) | 0.41 | 0.04  (-0.10, 0.18) | 0.55 | 0.03  (-0.14, 0.21) | 0.74 |
| Fiber, g/d | 0.05  (-0.09, 0.19) | 0.50 | -0.23  (-0.38, -0.09) | <0.01** | -0.25  (-0.39, -0.11) | <0.01** | -0.23  (-0.39, -0.06) | 0.01* |
| Glycemic Index, % | -0.09  (-0.24, 0.05) | 0.21 | 0.22  (0.07, 0.37) | <0.01** | 0.23  (0.08, 0.37) | <0.01** | 0.22  (0.06, 0.39) | 0.01* |
| Glycemic Load, units | -0.16  (-0.34, 0.03) | 0.10 | 0.26  (0.07, 0.44) | 0.01* | 0.25  (0.06, 0.43) | 0.01* | 0.30  (0.10, 0.49) | <0.01** |
| Whole-grain,  %Carbs | 0.06  (-0.08, 0.20) | 0.39 | -0.17  (-0.31, -0.03) | 0.02* | -0.18  (-0.32, -0.04) | 0.01* | -0.24  (-0.41, -0.09) | <0.01** |
| Refined-grain,  %Carbs | -0.07  (-0.21, 0.07) | 0.35 | 0.12  (-0.02, 0.27) | 0.09 | 0.14  (-0.01, 0.29) | 0.05 | 0.28  (0.12, 0.44) | <0.01** |

* *p*<0.05；***p*<0.01；

Mix-effect model.

Considering the participant’s ID as random effect and day as a nested random effect.

Adjusted for age, duration, BMI, total insulin dose, Insulin regimen, physical activity and other macro-nutrients listed.

3. *Design limitations must be acknowledged prominently in the abstract and conclusions.*

Response 3: We sincerely appreciate the reviewers’ insightful comments regarding the study design. We fully acknowledge that as a cross-sectional study, our research provides a lower level of evidence compared to randomized controlled trials (RCTs), which we have now explicitly addressed in both the abstract, discussion and conclusion sections:

**Abstract:**

Added the following limitation statement:

“However, as a cross-sectional analysis, these findings represent observational associations rather than causal evidence. Further validation through prospective cohorts and randomized trials is needed to assess clinical applicability.” ( line 40-42)

**Discussion:**

“Our study also has some limitations. First, it is a cross-sectional study, and all results are based on association analysis. Compared to randomized controlled clinical trials, the level of evidence is relatively weak. Second, our sample size was relatively small in comparison to other similar studies. The sample size reduces power to detect smaller effects and limits subgroup analyses. However, most previous studies primarily used self-blood glucose monitoring or HbA1c to evaluate blood glucose control. Apart from this, in a recent study of a large sample of T1D patients, dietary records were used to assess the impact of dietary factors while glycemic control was evaluated using CGM; However, the two assessments were not synchronized. Third, dietary data reliance on self-report may introduce measurement bias, though we mitigated this through standardized collection tools.” ( line 385-394 )

4. *Clinical recommendations inappropriate for observational data must be removed.*

Response 4: We are grateful to the reviewers for this valuable comment. Indeed, our cross-sectional design limits the ability to formulate clinical guidelines. Accordingly, we have implemented the following modifications:

In the abstract, we have removed the clinical recommendation “Increasing the consumption of dietary fiber is beneficial for achieving long-term glycemic control. Additionally, increasing the intake of whole grains, especially at lunch, as a source of high-quality carbohydrates, is helpful in reducing glycemic fluctuations, which is of crucial significance for reducing the risk of long-term complications in T1D patients.” and replaced it with statement “From a long-term glycemic control perspective, higher dietary fiber intake appears to be associated with improved HbA1c levels, while in terms of short-term glycemic variability, increased whole grain consumption is associated with reduced glucose fluctuations. However, as a cross-sectional analysis, these findings represent observational associations rather than causal evidence. Further validation through prospective cohorts and randomized trials is needed to assess clinical applicability.” ( line 38-43 ) to maintain appropriate focus on our study findings rather than clinical guidance.

In the Discussion section, we have replaced the original statement “For patients with T1D, increasing the consumption of whole grains, particularly at lunch, as a source of high-quality carbohydrates, is advantageous for reducing post-meal glycemic fluctuations when a low-carbohydrate diet pattern is not required” with “For patients with T1D, higher consumption of whole grains as a primary carbohydrate source - particularly during lunch - is associated with attenuated postprandial glycemic excursions when a low-carbohydrate diet pattern is not required.” (Line 382-384).

In the conclusion section, we have replaced the original statement “Increasing the consumption of dietary fiber is beneficial for achieving long-term glycemic control. Additionally, increasing the intake of whole grains, especially at lunch, as a source of high-quality carbohydrates, is helpful in reducing post-meal glycemic fluctuations, which is of crucial significance for reducing the risk of long-term complications in T1D patients” with “Evidence from this study indicates that elevated dietary fiber intake is significantly associated with enhanced long-term glycemic control in individuals with T1D. Notably, our findings reveal that preferential consumption of whole grains as the predominant carbohydrate source, with particular emphasis on lunchtime intake, is correlated with reduced postprandial glucose variability.” (Line 408-411)

5. *Complete individual-level datasets must be provided per PLOS policy requirements.*

Response 5: In full compliance with PLOS ONE's data sharing policy, all raw data underlying this study's findings are provided as Supporting Information files （Supporting information-original datasets）

***Reviewer #2:***

***Required Revisions:***

1. *The current title does not fully capture the scope and analytical depth of the study. While it accurately reflects the general finding, it overlooks key aspects such as the study design (cross-sectional), the use of both FFQ and 3-day meal-based dietary records, and the incorporation of CGM-derived glycemic variability metrics analyzed through mixed-effects and machine learning models.*

*I recommend revising the title to better reflect these methodological components and provide a clearer picture of the study at a glance. A more appropriate and informative title might be:*

*“Association Between Carbohydrate Quality and Glycemic Control in Individuals with Type 1 Diabetes: A Cross-Sectional and Meal-Based Analysis”*

*Or*

*“Carbohydrate Quality and Glycemic Outcomes in Type 1 Diabetes: Evidence from Cross-Sectional and Meal-Level Data Analyses”*

Response 1: We sincerely appreciate the reviewer's insightful suggestion regarding our original title. Upon reflection, we agree that it failed to adequately reflect both our study design and analytical approach. In direct response to this comment, we have revised the title to:

"Association Between Carbohydrate Quality and Glycemic Control in Individuals with Type 1 Diabetes: A Cross-Sectional and Meal-Based Analysis" ( line 1-3 )

We believe this revised title more accurately represents the scope and methodology of our work while maintaining clarity for readers.

2. *The association between higher dietary protein intake and elevated HbA1c levels (Tables 2 and 3) contrasts with existing literature that suggests protein intake is typically neutral or beneficial for glycemic control in T1D. The discussion should address possible explanations for this discrepancy, including residual confounding, differences in protein source (e.g., animal vs. plant), dietary context, or reverse causality (e.g., individuals with poor control increasing protein intake following clinical advice).*

Response 2: We are grateful to the reviewer for raising this important question about the protein-glycemic control association, which has prompted us to clarify several key aspects in our revision.

Our study revealed that habitual protein intake, estimated by FFQ, was positively associated with HbA1c, whereas short-term protein intake captured by 3-day weighed dietary records showed no relation to glycaemic variability. These data indicate that protein may exert its influence on long-term, rather than acute, glycaemic control in T1D. The literature on dietary protein and glycaemia remains inconsistent. In T1D, acute protein loads (>75 g) can trigger delayed post-prandial hyperglycaemia 3–5 h after ingestion, presumably via gluconeogenesis and glucagon-mediated mechanisms[1]. A recent RCT in T1D adolescents demonstrated that a high-protein meal (110 g protein, 70 g carbohydrate) produced larger 12h glycaemic excursions than a low-protein comparator (28 g protein, 70 g carbohydrate), highlighting the prolonged glycaemic footprint of protein[2]. A comprehensive review[3] emphasized that the glycaemic response depends not only on the quantity but also on the absorption kinetics and amino-acid composition of protein[4]. Whey, which remains soluble in gastric acid, empties rapidly, whereas casein coagulates and delays gastric emptying[5]. Specific amino acids—alanine, serine, glycine (via serine), cysteine and arginine—potentiate glucagon secretion[6], amplifying hepatic glucose output. Consequently, the glycaemic impact of protein in T1D is multifactorial and may be confounded by concurrent fat and carbohydrate intake. By contrast, meta-analyses in T2D (42 RCTs, n = 4 809) show that sustained high-protein diets improve fasting glucose (−0.89 mmol/L) through enhanced satiety (−200 to −300 kcal/day), preservation of lean mass and increased diet-induced thermogenesis (~10 % of total energy expenditure)[7]. Benefits on insulin sensitivity peaked at 6 months but attenuated by 12 months, reflecting waning adherence and metabolic adaptation. These divergent findings underscore pathophysiological differences between T1D and T2D, distinct glucoregulatory capacities and the pivotal role of exogenous insulin dosing in T1D. Our findings are constrained by the small sample size, recall bias inherent to FFQs and the cross-sectional design, precluding causal inference. Prospective cohort studies and rigorously controlled randomized trials with refined methodological approaches are required to delineate the true relationship between dietary protein and glycaemic outcomes in T1D.

We have incorporated this additional content into the Discussion section. ( line 351-375 )

References

1.	Smart, C.E., et al., *Both dietary protein and fat increase postprandial glucose excursions in children with type 1 diabetes, and the effect is additive.* Diabetes Care, 2013. **36**(12): p. 3897-902.

2.	Neu, A., et al., *Higher glucose concentrations following protein- and fat-rich meals - the Tuebingen Grill Study: a pilot study in adolescents with type 1 diabetes.* Pediatr Diabetes, 2015. **16**(8): p. 587-91.

3.	Dao, G.M., et al., *The Glycemic Impact of Protein Ingestion in People With Type 1 Diabetes.* Diabetes Care, 2025. **48**(4): p. 509-518.

4.	He, T. and M.L. Giuseppin, *Slow and fast dietary proteins differentially modulate postprandial metabolism.* Int J Food Sci Nutr, 2014. **65**(3): p. 386-90.

5.	Boutrou, R., et al., *Sequential release of milk protein-derived bioactive peptides in the jejunum in healthy humans.* Am J Clin Nutr, 2013. **97**(6): p. 1314-23.

6.	Calbet, J.A. and D.A. MacLean, *Plasma glucagon and insulin responses depend on the rate of appearance of amino acids after ingestion of different protein solutions in humans.* J Nutr, 2002. **132**(8): p. 2174-82.

7.	Jing, T., et al., *Effect of Dietary Approaches on Glycemic Control in Patients with Type 2 Diabetes: A Systematic Review with Network Meta-Analysis of Randomized Trials.* Nutrients, 2023. **15**(14).

3. *The interpretation of the MCA results suggests an association between high-quality carbohydrate intake and better glycemic control. It should be emphasized that these findings are observational and do not imply causality due to the cross-sectional design. Clarify whether the dietary variables independently contributed to quadrant clustering or whether other unmeasured factors may have influenced the patterns.*

Response 3: Thank you for this important observation.

Multiple Correspondence Analysis (MCA) analyzes associations between multiple categorical variables by reducing dimensionality (usually to 2D/3D) for visualizing category relationships[1]. Our study implemented MCA to detect the co-occurring glycaemic and dietary features in the T1D cohort. In the MCA, high-quality carbohydrate intake was associated with better glycemic control. However, as the reviewer pointed out, the MCA pattern merely reflects the co-occurrence of dietary and metabolic variables at one point in time, and these findings are observational and do not imply causality due to the cross-sectional design. We subsequently fitted mixed-effects models that adjusted for both dietary and demographic covariates and found whole-grain and dietary-fiber intakes to be significantly and inversely associated with glycaemic variability. Nevertheless, prospective cohorts and rigorous randomized controlled trials are still required to establish causality.

References

1.	Cherubini, V., et al., *Rethinking Carbohydrate Intake and Time in Range in Children and Adolescents with Type 1 Diabetes.* Nutrients, 2021. **13**(11).

4. *Misclassification bias should be acknowledged as a limitation. Despite using validated tools, self-reported dietary data—especially regarding portion sizes, meal timing, and food type classification—are subject to error and may affect the observed associations.*

Response 4: We fully acknowledge the limitations of dietary assessment methods raised by the reviewer.

We employed a FFQ that underwent rigorous testing for reliability (test-retest ICC = 0.85) and validity (r = 0.62 against 3-day dietary records) . In nutritional epidemiology research, FFQs are commonly utilized as a practical tool for collecting long-term dietary consumption data among large study populations.[1]

Although we implemented standardized training, recall bias and portion estimation errors remain unavoidable which we have acknowledged as a limitation in the Discussion. (line 392-394 )

In future studies, we aim to design precise investigations, such as analyzing dietary metabolites and conducting gut microbiota-related research, to more accurately assess whole grain and dietary fiber intake.

References

1.	Pokharel, P., et al., *Vegetable, but Not Potato, Intake Is Associated With a Lower Risk of Type 2 Diabetes in the Danish Diet, Cancer and Health Cohort.* Diabetes Care, 2023. **46**(2): p. 286-296.

5. *There is no table in the manuscript or supplementary materials that clearly presents the demographic and clinical characteristics of the study participants (e.g., age range, gender distribution, BMI, diabetes duration, insulin regimen). This information is essential for evaluating the generalizability and context of the findings. Please include a table in the main text or supplementary materials summarizing these baseline characteristics.*

Response 5: Due to the limitation on the number of figures/tables allowed in the main text, we have included the key results figures in the manuscript body while presenting the baseline characteristics of participants (including FFQ and 3-day 24-hour dietary records) as Supplementary Materials. These data are now available in Supplementary Table S1 (for FFQ) and Supplementary Table S2 (for 3 day dietary records). We would be grateful if the reviewer could kindly evaluate this supplementary information.

***Reviewer #3:***

***Required Revisions:***

1. *-line 29: Please replace "established" with "used" to describe the utilization of LightGBM model in the analysis*

Response1: We sincerely appreciate the reviewer's insightful comments. We have replaced “established” with “used” to describe the utilization of LightGBM model in the analysis (line 29).

2. *-line 77: Please replace "dietary intake" with "the quality of carbohydrates in the diet" which better describes the aim of the study*

Response 2: We sincerely appreciate the reviewer's insightful comments. We have replaced "dietary intake" with "the quality of carbohydrates in the diet" which better describes the aim of the study (line 79).

3. *-line 87: Exclusion criteria: Did the authors consider hypothyroidism specifically, given the association between hypothyroidism and Type 1 Diabetes?*

Response 3: We sincerely appreciate the reviewer’s insightful comments regarding the association between autoimmune diabetes and autoimmune thyroid disease (AITD). Because the present study focuses primarily on glycaemic outcomes in individuals with T1D and does not delve into immunological markers such as autoantibodies or β-cell function, we did not categorically exclude participants with autoimmune thyroid disorders. Nevertheless, patients with clinically unstable hyper- or hypothyroidism were excluded to minimize confounding, e.g., thyroid-stimulating hormone [TSH] levels <0.2 μIU/mL or >4.2 μIU/mL. We have elaborated this point in the inclusion/exclusion criteria section.(line 89-91)

4. *-lines 100 - 101: "Subsequently, a total of 65 patients maintained a 3-day food record..." Are there specific criteria for selecting the 65 participants who maintained a 3-day food record and wore the CGM? Please include the method of section in the manuscript.*

Response 4: We sincerely appreciate the reviewer's question regarding participant selection. The inclusion criteria for subjects completing the 3-day dietary records were as follows: 1) Individuals who consent to participate in the study and provide informed consent; 2) Age range: 10 to 65 years; 3) Confirmed diagnosis of T1D according to ADA2020 standards; 4) Requiring exogenous insulin therapy; 5) HbA1c level less than 12%; 6) Participants were required to wear CGM devices for 7 consecutive days and complete three weighed-food dietary records (2 weekdays + 1 weekend day) during the middle 5 days, including submission of food photographs to researchers. Exclusion criteria included pregnancy, celiac disease, and any other illness besides diabetes that significantly affected their health status. We have now incorporated the specific inclusion/exclusion criteria for the 3-day dietary record component into the Methods section.( line 91-94 )

5. *-line 101: mentioned that these 65 participants wore the CGM:How long were participants expected to wear the CGM? Did all participants wear the CGM for the same length of time? Please include this information in the manuscript.*

Response 5: We sincerely appreciate the reviewer's insightful question regarding our study protocol. In response, we have now explicitly detailed the continuous glucose monitoring (CGM) and dietary assessment methodology in the Methods section (line 118-123) as follows: The glycemic variability of T1D patients was assessed using a Continuous Glucose Monitor (Anytime CT15) for a standardized 7-day monitoring period. During the CGM period, participants completed three 24-hour dietary records，included 2 weekdays and 1 weekend day to capture habitual intake variations during the middle 5-day monitoring period to assess typical intake patterns, including precise portion measurements. At the end of the experimental period, data from CGM were downloaded by dedicated informatics platforms.

The 3-day dietary record, a widely used nutritional assessment method in research, offers high short-term accuracy with reduced recall bias. It captures detailed meal timing and cooking methods, making it particularly suitable for studies requiring precise short-term dietary data.[1, 2].

References

1.	Katz, M.L., et al., *Associations of nutrient intake with glycemic control in youth with type 1 diabetes: differences by insulin regimen.* Diabetes Technol Ther, 2014. **16**(8): p. 512-8.

2.	Hakola, L., et al., *Association of Cereal, Gluten, and Dietary Fiber Intake With Islet Autoimmunity and Type 1 Diabetes.* JAMA Pediatr, 2019. **173**(10): p. 953-960.

6. *-lines 119-124: described various analysis performed. Was there additional analysis to test for multicollinearity among the independent variables?*

Response 6: We sincerely appreciate the reviewer's insightful comment regarding multicollinearity, a crucial consideration when fitting regression models with dietary variables that may exhibit inherent intercorrelations. In our statistical analysis: Multicollinearity Assessment: Systematically evaluated all regression models using variance inflation factors (VIF). We Applied the conventional threshold of VIF < 5 as evidence against substantial multicollinearity. Model Diagnostics: All fitted regression models demonstrated VIF values < 5.
