# Supplementary material for: Association between carbohydrate quality and glycemic control in individuals with type 1 diabetes: A cross-sectional and meal-based analysis
Source: PLoS One. 2026 Feb 2;21(2):e0341915. doi: 10.1371/journal.pone.0341915 (PMC12863503; doi:10.1371/journal.pone.0341915)
Supplement: Attachment — Submitted filename: Response to reviewers-R2.docx [file pone.0341915.s005.docx]

Response to comments

We sincerely thank the editor and all reviewers for their valuable feedback that we have used to improve the quality of our manuscript. The reviewers’ comments are laid out below in italicized font and specific concerns have been numbered. Our response is given in red font and changes to the manuscript are highlighted in yellow. The highlighted modified sections in the text correspond to the line numbers of the revised manuscript-R2- (clean copy) version.

Reviewer #1: Overall Assessment: This manuscript presents a well-designed observational study examining the association between carbohydrate quality and glycemic control in individuals with Type 1 diabetes. The authors have addressed previous reviewer concerns and the work makes a meaningful contribution to diabetes nutrition research.
***Language Issues Requiring Correction:***
*1. Line 119-120: "During the CGM period, participants completed three 24-hour dietary records，included 2 weekdays and 1 weekend day" - Replace Chinese comma with semicolon and change "included" to "including"*

Response 1. Thank you for pointing this out. We have revised the sentence as suggested: "During the CGM period, participants completed three 24-hour dietary records; including 2 weekdays and 1 weekend day."

*2. Line 367: Keep "**HbA1c" formatting consistent throughout (avoid subscript variations)*

Response 2. We appreciate you catching this inconsistency. We have double-checked the entire manuscript to ensure "HbA1c" is now presented consistently without subscript variations.

*3. General: Replace remaining Chinese punctuation marks (，) with standard English punctuation*

Response 3. We appreciate you highlighting this issue. We have conducted a global search and manual check to ensure that all Chinese punctuation marks have been replaced with their standard English equivalents throughout the document.

***Scientific Comments:***
*1. Effect sizes: While statistically significant, some associations (e.g., fiber OR=1.101) are modest. The clinical significance could be better explained.*

Response 1. We thank the reviewer for this insightful comment. We agree that while the association for dietary fiber is statistically significant, its modest effect size (OR=1.101) warrants a careful discussion of its clinical and public health relevance. We have addressed this nuance in the revised manuscript (Discussion section, line 342-352), and our interpretation is outlined below.

In this study, dietary fiber intake demonstrated a significant positive association with achieving glycemic targets (OR = 1.101, 95% CI: 1.009–1.201). Although the effect size may seem limited, its public health relevance should not be underestimated. An OR of 1.101 corresponds to a 10.1% increase in the odds of meeting glycemic targets for each additional gram of daily dietary fiber intake. This dose-response relationship suggests that sustained, incremental increases in fiber consumption may offer meaningful auxiliary benefits for glycemic management. These findings are consistent with the known physiological effects of dietary fiber, including delayed gastric emptying and attenuation of postprandial glucose excursions.

It is noteworthy that this association remained statistically significant even after adjustment for clinically influential covariates such as diabetes duration, insulin dose, and total energy intake, underscoring the independent contribution of dietary fiber. That said, the modest effect size indicates that dietary fiber should be viewed as a valuable component within a multifaceted dietary approach rather than a standalone intervention in T1D management. Encouraging fiber-rich dietary patterns could contribute meaningfully to improving population-level glycemic control outcomes.

*2. Lunch findings: The observation that glycemic variability is highest at lunch is interesting and deserves further investigation in future studies.*

Response 2. Thank you for this positive feedback and for highlighting the interest of this finding. As we mentioned in the discussion (line 387-391), Our findings indicate that patients with T1D experience significant post-lunch blood glucose fluctuations, commonly known as the "dusk phenomenon". Therefore, choosing foods with low GI and GL such as increasing the proportion of whole grains in carbohydrate sources during this time may help mitigate these fluctuations. We agree that the pronounced glycemic variability observed during the lunch period is an intriguing result that merits deeper exploration. Further studies are warranted to elucidate the underlying mechanisms of this phenomenon, particularly the roles of lunch-specific meal composition, meal timing, circadian rhythms, and physical activity patterns.

We have incorporated the above suggestions in the Discussion section (line 394-398) to reflect this profound insight.

*3. Study population: Results from this Chinese population may not apply directly to other ethnic groups. This limitation is properly acknowledged.*

Response 3. We appreciate the reviewer's comment regarding the generalizability of our results. We fully agree that findings from our Chinese cohort may not be directly extrapolated to other ethnic populations due to potential differences in genetic background, diet, lifestyle, and healthcare environments. We have explicitly stated this as a key limitation in the Discussion section (line 422-424), and we thank the reviewer for validating its importance.

***Suggestions:***
*1. Consider discussing the practical challenges of increasing whole grain consumption in this population.*

Response 1. We thank the reviewer for raising this practical point. We agree that a discussion of the implementation challenges is essential for contextualizing our findings within real-world clinical and lifestyle management. In response to this comment, we have added a new paragraph in the Discussion section (line 425-433) addressing the specific practical and behavioral barriers to increasing whole grain intake in individuals with T1D.

Despite the observed benefits, translating these findings into practice faces several challenges specific to the T1D population. A primary concern is the practical difficulty of accurately estimating the carbohydrate content of whole grain foods for insulin dosing, as their high fiber content can affect glycemic response and necessitate individualized insulin dose adjustments. Furthermore, initial gastrointestinal discomfort, suboptimal palatability, limited availability, and deeply ingrained dietary habits collectively pose significant barriers to the adherence and acceptance of whole-grain products. Therefore, any recommendation to increase whole grain intake should be accompanied by structured nutritional education, close glucose monitoring, and collaboration between patients and dietitians to tailor changes to individual lifestyles and insulin regimens.
*2. The protein-HbA1c association, while well-explained, could benefit from additional investigation in future work.*

Response 2. We thank the reviewer for their positive assessment of our explanation and for this constructive suggestion. We agree that further investigation into the relationship between dietary protein intake and HbA1c in T1D would be valuable for clarifying its clinical relevance. Further research is needed to clarify whether this relationship is influenced by protein type (e.g., whey, casein, soy, or gluten), meal timing, or concomitant fat and carbohydrate content. Intervention trials using isoenergetic protein substitutions could help determine causal effects and inform practical dietary recommendations. *3. Future studies might use biochemical markers (e.g., alkylresorcinols) to validate self-reported whole grain intake.*
Response 3. We sincerely thank the reviewer for raising this valuable methodological point. In response to this comment, we have added a new paragraph in the Discussion section (line 418-422). We fully agree that incorporating objective biomarkers would significantly strengthen the validity of dietary intake assessment. Self-reported measures, while practical for large-scale studies, are susceptible to recall bias and measurement error. The use of alkylresorcinols—which provide an objective, quantitative measure of whole grain wheat and rye intake over the preceding 1-2 days—would not only help validate food frequency questionnaires or dietary records but also enable more precise classification of exposure in future analyses. Future studies should seek to integrate objective biomarkers of dietary intake—such as plasma or urinary alkylresorcinols as a specific marker of whole grain wheat and rye consumption—to complement and validate self-reported data, thereby reducing misclassification and improving the accuracy of nutritional assessments in relation to glycemic outcomes.

*Reviewer #3: I commend the authors' work. Thank you for addressing previous comments and for the detailed response.*

Response 3. We are very grateful to the reviewers for the kind words and positive feedback. We appreciate the time and effort they have invested in reviewing our work.
