# Supplementary material for: Association between carbohydrate quality and glycemic control in individuals with type 1 diabetes: A cross-sectional and meal-based analysis
Source: PLoS One. 2026 Feb 2;21(2):e0341915. doi: 10.1371/journal.pone.0341915 (PMC12863503; doi:10.1371/journal.pone.0341915)
Supplement: Attachment — Submitted filename: Response to comments-R3.docx [file pone.0341915.s006.docx]

Response to comments

We sincerely thank the editor and all reviewers for their valuable feedback that we have used to improve the quality of our manuscript. The reviewers’ comments are laid out below in italicized font and specific concerns have been numbered. Our response is given in red font and changes to the manuscript are highlighted in yellow. The highlighted modified sections in the text correspond to the line numbers of the Revised Manuscript (clean copy) version.

**Reviewer #1:** This manuscript examines carbohydrate quality and glycemic control in Type 1 diabetes using food frequency questionnaires and synchronized dietary records with CGM. The research question is clinically relevant, but critical statistical issues require correction before publication.

***Critical Issues***

1. *Multiple Testing: The analysis tests approximately 8 dietary variables × 5 outcomes × 3 meals without correction for multiple comparisons. With approximately 120 tests at α=0.05, approximately 6 false positives are expected by chance alone. The authors must either apply Bonferroni, Holm, or false discovery rate correction, or explicitly acknowledge this as exploratory hypothesis-generating research requiring confirmation. Several associations with p-values between 0.01-0.05 may be statistical artifacts.*

Response 1: We thank the reviewer for this critical and valid point regarding multiple testing. We completely agree that conducting approximately 120 statistical tests without correction substantially increases the risk of false positives, and that several associations with p-values in the 0.01-0.05 range could indeed be statistical artifacts.

In response, we have revised the manuscript to explicitly frame this analysis as exploratory and hypothesis-generating. In the Statistical Analysis section of the Methods, we have now added the following statement (lines 155-157): "Given the large number of statistical tests performed in this exploratory analysis, the findings, particularly those with p-values between 0.01 and 0.05, should be interpreted with caution and require independent confirmation in future studies."

Furthermore, in the Results sections, we have toned down the language surrounding these marginally significant associations (lines 236-241), emphasizing their preliminary nature and referring to them as "suggestive of an association." We believe this approach transparently acknowledges the limitation raised by the reviewer while still allowing the presentation of potential patterns for future research.

In addition, for the pre-specified primary comparisons (e.g., glycemic control and nutrient distribution across three meals), these were treated as confirmatory analyses. These analyses employed linear mixed-effects models, with Bonferroni correction applied for post-hoc testing to strictly control the type I error rate (Table 5). For the extensive analyses exploring relationships between dietary variables and multiple glucose outcomes, these were defined as hypothesis-generating analyses. Therefore, to avoid excessive conservatism and retain potentially biologically meaningful preliminary signals, no global multiple comparison correction was applied to this set of analyses. We have clearly stated in the "Conclusions" sections of the manuscript that these findings are preliminary and explanatory in nature, serving primarily to generate hypotheses for future research and require validation in independent cohorts. (lines 449-450)

2. *LightGBM Validation: R²=95-96% suggests potential overfitting. The manuscript provides no cross-validation details (split-sample? k-fold? leave-one-out?). With only 65 subjects and 17 features, overfitting is highly likely. The authors must document proper validation methodology or remove this section. The SHAP analysis conclusions cannot be trusted without demonstrated generalization.*

Response 2: We thank the reviewer for this critical assessment of the LightGBM model. We fully agree with the reviewer's assessment. The reported R² of 95-96% is highly suggestive of overfitting, given our limited sample size of 65 participants and the absence of a robust external validation cohort. We acknowledge that the initial submission lacked the necessary cross-validation details to demonstrate the model's generalizability, and as a result, the associated SHAP analysis conclusions cannot be considered reliable.

In light of these valid concerns and the inherent difficulty in providing a convincingly generalizable model with the current dataset, we have decided to remove the entire section concerning the LightGBM model and SHAP analysis from the revised manuscript.

Importantly, upon careful reconsideration, we have confirmed that removing this machine learning component does not affect the main conclusions of our study, which are robustly supported by the primary mixed-effects models examining the associations between dietary factors and glycemic outcomes. This action therefore strengthens the manuscript by eliminating potentially misleading results and allowing us to focus the narrative on our core, statistically sound findings. We are grateful to the reviewer for their insightful comments, which have helped us improve the overall quality and reliability of our work.

3. *Mixed-Effects Specification: The manuscript states mixed-effects models were used but doesn't fully specify random effects structure. For nested data (meals within days within persons), the authors must state whether random slopes are included, what correlation structure is assumed, and provide complete model specifications.*

Response 3: We thank the reviewer for highlighting this omission in our model specification. We have now provided a comprehensive description of the mixed-effects models in the revised manuscript to ensure clarity and reproducibility (lines 145-151).

1. Random Effects Structure:

Our models included random intercepts to account for the nested structure of the data and the non-independence of repeated measures. Specifically, we included: A random intercept for Subject ID to capture baseline differences between individuals. A random intercept for Day nested within Subject ID to capture day-to-day variability within the same individual.

2. Random Slopes and Correlation Structure:

Given that the study aimed to estimate average effects at the population level (not individual heterogeneity), combined with the limited sample size (65 participants contributing 188 days of dietary records), we did not include any random slopes for the dietary variables. We assumed that the fixed effects of the dietary variables are consistent across all subjects and days.

3. Complete Model Specification:

The model for a given outcome variable (TIR) can be written as:

TIR_{ij} = β₀ + β₁Ekcal_{ij} + β₂FatE_{ij} + β_3_ProE_{ij} + β_4_Fiberg _{ij} + β_5_ Duration_{ij} + β_6_Age_{ij} + β_7_BMI_{ij} + β_8_InsulinDose_{ij} + β_9_PhysicalActivity _{ij} + β_10_InsulinRegimen + u i + v i(j) + ε_{ij}

Where:

TIR_{ij} is the Time in Range for subject i on day j.

Fixed effects: β_0_ is the overall intercept; β_1_, …, β_10_ are coefficients for the predictors: total energy intake (Ekcal), fat energy proportion (FatE), protein energy proportion (ProE), fiber intake (Fiberg), diabetes duration (Duration), age (Age), body mass index (BMI), insulin dose, physical activity level, and insulin regimen (Insulin_Regimen).

u i is the random intercept for subject i.

v i(j) is the random intercept for day j nested within subject i

ε_{ij} is the residual error term.

4. *Unmeasured Confounding: The models omit insulin delivery method, carbohydrate counting accuracy, hypoglycemia treatment behaviors, and socioeconomic status—all plausible confounders of observed associations. A discussion paragraph explicitly acknowledging these unmeasured confounders is required.*

Response 4: We thank the reviewer for this insightful comment. We fully agree that unmeasured confounding is a fundamental limitation of any observational study, and the factors mentioned by the reviewer (insulin delivery method, carbohydrate counting accuracy, hypoglycemia treatment behaviors, and socioeconomic status) are indeed plausible confounders of the association between carbohydrate quality and glycemic outcomes. Among these confounders, insulin delivery method was specifically included in the models as a covariate to adjust for its direct and critical impact on glycemic outcomes. Other potential confounding factors, which were not adjusted for in the models, are addressed in the Discussion section.

In response to this comment, we have taken the following actions:

We have added a dedicated paragraph in the Discussion section, to explicitly and thoroughly address this issue. Insulin delivery method was included in the models as a covariate to adjust for its potential effect on glycemic outcomes (lines 421-428). The new text reads:

"Most importantly, as an observational study, it is susceptible to unmeasured confounding. Factors such as carbohydrate counting accuracy, hypoglycemia management behaviors, and socioeconomic status were not measured in this analysis. These factors could influence both dietary choices and glycemic control (HbA1c), and thus may partly account for the observed associations. For instance, individuals with higher health literacy or socioeconomic status might simultaneously have better access to high-fiber foods and achieve better glycemic management. Future studies that meticulously collect data on these potential confounders are needed to confirm the magnitude and independence of these dietary effects.

5. *Missing Data: Line 148 mentions "multiple imputation" with no supporting details. The manuscript must specify which variables had missing data, what percentage was missing, how many imputations were performed, and what imputation model was used.*

Response 5: We thank the reviewer for pointing out the need for details on our handling of missing data. A table summarizing the missing data statistics for all variables has been included as part of the supplementary materials (Supporting information-R3 S3-S5 Table). We have now comprehensively addressed this in the revised manuscript within the “Statistical Analysis” section (lines 158-164). Our approach was as follows:

We use Multiple Imputation by Chained Equations (MICE) under the assumption that the data were missing at random (MAR). The imputation was performed using the mice package in R. We generated m = 50 imputed datasets to ensure the efficiency of the estimates. The imputation model included all variables used in the primary analysis as well as auxiliary variables that were predictive of the missingness or the missing values themselves. The algorithm ran for 20 iterations. All statistical models were fitted to each of the 50 imputed datasets, and the results were pooled into a single set of estimates using Rubin's rules.

6. *Sample Size: With only 65 participants for 3-day records and 188 lunch observations, statistical power is limited for several analyses. The authors should quantify which specific analyses are underpowered, as null findings may reflect insufficient power rather than true absence of effects.*

Response 6: We thank the reviewer for this critical comment regarding statistical power. We fully agree that the null finding for the association between protein energy proportion at lunch and postprandial glycemic variability should be interpreted with caution, as it may reflect a Type II error rather than a true absence of effect.

To directly address this, we performed a post hoc power analysis for this specific analysis. The analysis was based on the following parameters:

Outcome: Glycemic variability (e.g., MAGE) at the meal level. Sample Size: n = 188 lunch observations. Significance Level (α): 0.05. Effect size f²: f² = R² / (1 - R²)，R²≈0.01，f² ≈ (0.1)² / (1 - (0.1)²) = 0.01 / 0.99 ≈ 0.01. The analysis revealed that our statistical power to detect an effect of this size was approximately 14%. This means that if a true, modest association between lunchtime protein intake and glycemic variability exists in the broader population, our study had only a 14% probability of correctly identifying it as statistically significant. Conversely, there was an 86% probability of missing it (Type II error).

We have added a dedicated section in the Discussion that explicitly states (lines 428-433):

“Finally, the statistical power of this study was limited by the sample size. Although it was adequate for detecting moderate-to-large effects, the study was likely underpowered to identify smaller, yet potentially important, associations. For instance, the non-significant finding for the association between protein energy proportion at lunch and postprandial glycemic variability should be interpreted with caution, as it may reflect a Type II error rather than conclusive evidence for the absence of an effect.”

***Moderate Issues***

1. *Protein Discussion: Lines 361-385 provide extensive mechanistic speculation beyond what the cross-sectional data can support. Reverse causation (poor glycemic control leading to altered protein intake) is equally plausible but not adequately discussed. This section should be condensed or alternative explanations presented with equal weight.*

Response 1: We thank the reviewer for this critical observation. We agree that the previous version of the discussion contained excessive mechanistic speculation that was not fully supported by our cross-sectional data.

In direct response to this comment, we have thoroughly revised the section (Lines 361-385, now corresponding to Lines (lines 362-373) in the revised manuscript) as follows:

It is worth noting that our study suggested a potential differential effect of dietary protein in T1D: habitual intake (FFQ) was associated with long-term glycaemic control (HbA1c), while short-term intake (3-day records) showed no link to acute variability. This aligns with evidence that large acute protein loads can induce delayed hyperglycaemia via gluconeogenesis and glucagon secretion [39] [40], yet the effect is multifactorial, influenced by protein type, co-ingested nutrients, and insulin dosing [41] [42] [43]. This contrasts with T2D, where high-protein diets often improve glycaemia [44]. Thus, the glycaemic impact of protein in T1D appears complex and distinct from T2D, warranting further investigation to inform tailored dietary advice. However, our findings are constrained by the small sample size, recall bias inherent to FFQs and the cross-sectional design, precluding causal inference. Prospective cohort studies and rigorously controlled randomized trials with refined methodological approaches are required to delineate the true relationship between dietary protein and glycaemic outcomes in T1D.

2. *Whole Grain Classification: The methodology for categorizing foods as whole versus refined grain is not described. Whether based on composition tables, package labels, or participant self-report substantially affects interpretation. Misclassification of mixed-grain products could drive observed associations.*

Response 2: Thank you for this important reminder. We fully agree that without an explicit definition for “whole-grain” vs. “refined-grain” foods, observational findings could easily be biased by misclassification. In response, we have added a new subsection entitled “Whole-grain classification” in the revised Methods (lines 112-121).

1) The classification of grain foods was primarily based on the “Food Composition in China (2009)”;
2) A food item was classified as a "whole grain" if whole grains were listed as the first ingredient on its packaged food label (as per the database) or, for non-packaged items, if it was explicitly defined as such in the database (e.g., brown rice, oatmeal, whole-wheat bread). Conversely, items were classified as "refined grain" if the primary grain ingredient was not a whole grain (e.g., white bread, white rice, pasta made from refined flour).
3) For mixed-grain products (e.g., breads containing a blend of whole-wheat and white flour), we relied on the database's classification and the ingredient list hierarchy. If whole grains were not the first ingredient, the item was classified as refined grain.
4) The quantity of whole grains consumed was estimated based on the portion size reported by the participants and the proportional content provided by the food composition database.
3. *Effect Size Interpretation: OR=1.101 for fiber would imply implausibly large effects across the full intake range from current (10.3g) to recommended (25-36g) levels. The discussion should more explicitly address non-linearity possibilities and residual confounding.*

Response 3: We thank the reviewer for this exceptionally insightful comment. We agree that linearly extrapolating the observed odds ratio (OR) for fiber (1.101 per gram) across the entire range from current to recommended intake levels would imply an implausibly large effect, and we appreciate the opportunity to clarify and refine our interpretation.

We have modified the portion of the discussion (lines 347-353) concerning the interpretation of the dietary fiber effect size in the following manner:

“Crucially, the association between dietary fiber and glycemic outcomes (OR=1.101) must be contextualized by the low median fiber intake (10.32g/d) in our cohort. Therefore, a linear extrapolation of this effect to the recommended daily intake (25-36g/d) is not appropriate, as the dose-response relationship may be non-linear and exhibit diminishing returns at higher levels. In addition, as with any observational study, we cannot rule out the possibility of residual confounding due to unmeasured or imperfectly measured factors, such as overall dietary quality, socioeconomic status, or other healthy lifestyle behaviors associated with higher fiber intake.”

4. *Lunch Findings: The observation that lunch shows highest glycemic variability despite having highest fiber intake (alongside highest GI/GL) represents an internal inconsistency deserving discussion. Alternative explanations including diurnal insulin sensitivity patterns, meal location, or bolus timing should be considered.*

Response 4: We thank the reviewer for this insightful observation and for suggesting compelling alternative explanations. We agree that the co-occurrence of the highest glycemic variability with the highest fiber intake at lunch represents a fascinating and seemingly paradoxical finding that deserves a thorough discussion (lines 387-395). We have now expanded our discussion to address this point directly.

“An intriguing finding was the greatest glycemic variability at lunch, despite its highest fiber intake. This paradox underscores the multifactorial nature of glycemic control. Several factors may explain this: 1) the postprandial response is shaped by more than fiber, as co-ingested protein and fat can delay gastric emptying and prolong glycemic fluctuations[45]; 2) diurnal insulin resistance in the afternoon may blunt fiber's benefit; 3) the concurrently high meal GI/GL likely offset the high fiber's advantage by presenting a greater overall carbohydrate challenge; and 4) behavioral factors like bolus timing accuracy, particularly Automated Insulin Delivery (AID) systems[46], may differ at lunch. Consequently, nutritional advice should consider the overall meal context and concomitant behaviors, rather than focusing on single nutrients.”

***Minor Corrections***

1. Line 120: "records; including" should be "records, including" (incorrect semicolon usage)
   Line 299: "Fragile diabetes stage" is non-standard terminology—define clearly or use standard terms

Response 1: We thank the reviewer for their careful reading and for pointing out these issues with language and terminology. We have addressed them as follows in the revised manuscript:

Revised Line 86(Semicolon usage): The incorrect semicolon has been corrected to a comma. The sentence now reads: "...dietary records, including..."

Revised Line 298 ("Fragile diabetes stage"): We agree that this is a non-standard term. We have replaced it with a more standard and clinically descriptive phrase. It now reads: “Individuals with T1D can enter a stage of extreme glycemic instability following islet failure. This phase is characterized by severe glycemic lability and recurrent hypoglycemia, which lead to profound glucose fluctuations and the imperative for lifelong, carefully managed insulin replacement therapy.”

2. Lines 418-422: This 85-word sentence should be broken into 2-3 shorter sentences
   A data dictionary describing contents of datasets.zip would improve data reusability
   Consider adding subheadings in results section for improved organization

Response 2: We thank the reviewer for these constructive suggestions to improve the clarity and reusability of our manuscript. We have addressed all points as follows:

1) Long Sentence: The 85-word sentence has been broken down into three shorter, more readable sentences in the revised manuscript (lines 413-419). It now reads: “Future studies should aim to integrate objective biomarkers of dietary intake. For instance, plasma or urinary alkylresorcinols, which are specific markers of whole-grain wheat and rye consumption, can be used to complement and validate self-reported data. This approach would reduce misclassification and improve the accuracy of nutritional assessments in relation to glycemic outcomes.”
2) Data Dictionary: We have now created and uploaded a comprehensive data dictionary file (Data_Dictionary.docx).
3) Subheadings in Results: We have introduced clear subheadings within the Results section (e.g., " Dietary Fiber and Glycemic Control", " Short-Term Diet-Glycemia Relationship") to improve the organization and guide the reader through our findings.

***Summary***
The methodology is generally appropriate for observational research and the synchronized dietary-CGM approach represents a strength. However, statistical deficiencies—particularly the multiple testing problem and inadequate model validation—substantially undermine confidence in specific findings. These issues are correctable through revised methods and discussion sections rather than requiring new analyses. With appropriate statistical corrections and appropriately tempered conclusions, this manuscript would represent solid hypothesis-generating research for future intervention trials.

**Reviewer #4:** Dear Author

The most critical limitations and methodological flaws of your study are summarized concisely below:

1. Cross-Sectional Design (No Causation): The study design only permits the establishment of an association between carbohydrate quality (fiber/whole grains) and glycemic control {HbA1c). It cannot prove causality; the direction of the relationship is unclear.

Response 1: We thank the reviewer for this critical comment. We completely agree that the cross-sectional nature of our study design is an inherent limitation that prevents any causal inference regarding the relationship between carbohydrate quality and glycemic control.

We have also mentioned this point in the limitations section of our Discussion. It is anticipated that these correlational findings will provide a valuable foundation for future research by generating specific hypotheses to be tested in longitudinal cohort studies and randomized controlled trials.

2. Reliance on Self-Reported Data: Dietary intake, assessed via FFQs and 3-day records, is prone to recall bias and measurement error. Patients may misreport consumption, potentially exaggerating the health benefits observed.

Response 2: We thank the reviewer for raising this crucial point regarding the inherent limitations of self-reported dietary data. We fully acknowledge that food frequency questionnaires (FFQs) and dietary records are susceptible to measurement error and recall bias, which may lead to misclassification of participants' true dietary intake.

We mentioned in the Discussion section that (lines 403-413) “Third, dietary data reliance on self-report may introduce measurement bias, though we mitigated this through standardized collection tools. Our advantage lies in the fact that patients wore CGM while recording their diet, allowing us to obtain rich and detailed blood glucose data which is more suitable for studying the relationship between diet and blood glucose in T1D patients. In addition, there is a certain degree of recall bias in the FFQ; However, the subsequent 3-day diet recording is currently considered the gold standard for evaluating dietary composition. The combination of these two methods can enhance the reliability of our conclusions. Furthermore, all dietary records are completed under the guidance of a professional dietitian and meticulously checked to minimize errors. However, although we implemented standardized training, recall bias and portion estimation errors remain unavoidable.”

Despite these limitations, self-reported dietary instruments remain the cornerstone of nutritional epidemiology for assessing habitual intake in free-living populations, and the associations we observed are biologically plausible.

Future studies should aim to integrate objective biomarkers of dietary intake. For instance, plasma or urinary alkylresorcinols, which are specific markers of whole-grain wheat and rye consumption, can be used to complement and validate self-reported data. This approach would reduce misclassification and improve the accuracy of nutritional assessments in relation to glycemic outcomes.

3. Lack of Objective Validation: The study did not utilize objective biomarkers (such as alkylresorcinols) to confirm the self-reported whole grain intake, which is a key limitation in precisely assessing the dietary exposure.

Response 3: We thank the reviewer for this insightful comment. We fully agree that the lack of objective biomarkers to validate self-reported whole grain intake is a significant limitation in our study, as it introduces potential measurement error and prevents an absolutely precise assessment of dietary exposure.

In the “Limitations” section of the Discussion, we metioned “However, although we implemented standardized training, recall bias and portion estimation errors remain unavoidable. Besides this, future studies with larger sample sizes or well-designed randomised controlled trials are still needed to further clarify the relationship between carbohydrate quality and glycaemic control in individuals with T1D. Future studies should aim to integrate objective biomarkers of dietary intake. For instance, plasma or urinary alkylresorcinols, which are specific markers of whole-grain wheat and rye consumption, can be used to complement and validate self-reported data. This approach would reduce misclassification and improve the accuracy of nutritional assessments in relation to glycemic outcomes.” (lines 411-419)

Notwithstanding this limitation, our study provides valuable insights into the associations between carbohydrate quality and glycemic outcomes using tools that are feasible for application in large-scale, free-living populations, and the findings generate important hypotheses for future research. We believe that by transparently acknowledging this limitation and using it to chart a clear path for future research, we strengthen the scholarly contribution of our work. We are grateful to the reviewer for this valuable suggestion.

4. Limited Generalizability: The findings may be restricted to the specific ethnic/regional population studied and might not be directly applicable to Type 1 Diabetes patients in other parts of the world with different dietary habits.

Response 4: We thank the reviewer for this important comment regarding the generalizability of our findings. We acknowledge that our findings, derived from a cohort in Jiangsu Province, China, may have limited generalizability to the global T1D population due to regional variations in diet, culture, and lifestyle. Accordingly, we have cautioned against the direct extrapolation of our results to other ethnic groups. Nonetheless, Jiangsu represents a typical Eastern dietary pattern, which holds relevance for broader Chinese and East Asian contexts. Importantly, while the specific food sources of dietary fiber may vary across regions, the fundamental role of fiber intake remains universally significant. This is because culturally appropriate, high-fiber food options are accessible in most dietary patterns, underscoring the potential wider relevance of our observations. Accordingly, we have addressed this limitation in the Discussion section “Notably, owing to potential variations in genetic background, diet, lifestyle, and healthcare systems, the findings from our Chinese cohort should be extrapolated with caution to other ethnic populations.” (lines 419-421)

We thank the reviewer for helping us strengthen this aspect of our manuscript.

**Reviewer #5:** The present study investigates real-world data on dietary habits and their influence on glucose profiles in individuals with type 1 diabetes (T1D). The topic is relevant and the study is methodologically sound. However, several minor revisions would further improve the clarity and overall scientific quality of the manuscript:

1. The term “patients” should be avoided to ensure the use of inclusive language throughout the text. Please replace it with consistent terms such as “individuals,” “people,” or “participants.”

Response 1: We thank the reviewer for this important comment regarding the use of inclusive and person-first language. We fully agree with this principle and appreciate the opportunity to improve our manuscript.

In response, we have systematically replaced the term "patients" throughout the entire manuscript with more appropriate terms such as "individuals," and "participants," depending on the context.

2. Lines 65–68: The sentence appears confusing. A long-term low-carbohydrate diet cannot increase the risk of diabetes onset in individuals already diagnosed with T1D. Please revise this statement for accuracy and clarity.

Response 2: We thank the reviewer for this critical observation. We completely agree that the original statement was logically flawed and inaccurate, as individuals already diagnosed with T1D cannot be at risk for its onset. We apologize for this error.

Our intended meaning was to discuss the potential long-term risks associated with a sustained low-carbohydrate dietary pattern in this population, rather than the risk of onset.

We have therefore deleted the confusing sentence and replaced it with the following more accurate statement in the revised manuscript (Lines 63-66):

“The long-term safety of low-carbohydrate diets in T1D is controversial. While they may aid short-term glycemic control, some evidence suggests that sustained, very low-carbohydrate intake could be associated with increased mortality risk.”

3. Line 82: The sentence “155 patients diagnosed with T1D were enrolled” should appear only in the Results section. Additionally, sentences should not begin with Arabic numerals.

Response 3: We thank the reviewer for pointing out these issues regarding the manuscript structure and formatting conventions. We have addressed them as follows in the revised manuscript.

The sentence “155 patients diagnosed with T1D were enrolled” has been revised as “Individuals with T1D were enrolled”.

In the Results section, we described “From July 2022 to August 2024, a total of 155 T1D individuals from the specialized cohort for T1D at Jiangsu Province Hospital completed FFQ”.

4. To strengthen the scientific depth of the discussion, the authors are encouraged to elaborate on the following aspects:

The influence of major factors affecting postprandial glucose levels and metabolism, particularly the role of macronutrients and other dietary and lifestyle factors (doi: 10.1038/s41430-023-01359-8);
The association between daily carbohydrate (CHO) entries and glycemic outcomes, especially among users of automated insulin delivery systems (doi: 10.2337/dc25-0283).

Response 4: We sincerely thank the reviewer for these constructive and highly relevant suggestions to deepen our discussion. We have carefully studied the provided references and have expanded the Discussion section to incorporate these key aspects. (lines 387-395)

“An intriguing finding was the greatest glycemic variability at lunch, despite its highest fiber intake. This paradox underscores the multifactorial nature of glycemic control. Several factors may explain this: 1) the postprandial response is shaped by more than fiber, as co-ingested protein and fat can delay gastric emptying and prolong glycemic fluctuations[46]; 2) diurnal insulin resistance in the afternoon may blunt fiber's benefit; 3) the concurrently high meal GI/GL likely offset the high fiber's advantage by presenting a greater overall carbohydrate challenge; and 4) behavioral factors like bolus timing accuracy, particularly Automated Insulin Delivery (AID) systems[47], may differ at lunch. Consequently, nutritional advice should consider the overall meal context and concomitant behaviors, rather than focusing on single nutrients.”

5. Some figures (e.g., Figures 2b, 2c, and 5) appear blurry. Please improve image resolution and graphical quality to enhance readability.

Response 5: We thank the reviewer for pointing out the issue with the image resolution in Figures 2b, 2c, and 5. We apologize for this oversight in the initial submission.

In response, we have re-generated all figures using high-resolution source data and exported them in a suitable format to ensure optimal clarity and sharpness in both online and print formats.
